# Athlete Health Implications of Match Injuries in Portuguese Rugby Union

**DOI:** 10.3390/ijerph21091191

**Published:** 2024-09-08

**Authors:** Nuno Pinto de Sousa Lopes, António Miguel Cruz-Ferreira, Diana Torres Lima, Marta Amaral Silva, Luiz Miguel Santiago

**Affiliations:** 1 Department of Physical Medicine and Rehabilitation, Unidade Local de Saude de Sao José, R. da Beneficência 8, 1069-166 Lisboa, Portugal; 2 Federação Portuguesa de Rugby, R. Julieta Ferrão 12, 1600-007 Lisboa, Portugal; 3 Nova Medical School, Universidade Nova de Lisboa, Campo dos Mártires da Pátria 130, 1169-056 Lisboa, Portugal; 4 Sports Medicine Department, Portuguese Institute of Sports and Youth, 1649-028 Lisboa, Portugal; 5 Faculdade de Medicina, Universidade de Coimbra, Azinhaga de Santa Comba, Celas, 3000-548 Coimbra, Portugal; 6 Centro de Estudo e Investigação em Saúde, Faculdade de Economia, Universidade de Coimbra (CEISUC), 3004-512 Coimbra, Portugal

**Keywords:** sports medicine, injury surveillance, rugby, injury prevention, incidence

## Abstract

Objective: Our aim was to report time-loss match injuries in Portugal’s “Divisão de Honra” (Portuguese first tier) in the 2022/2023 season, focusing on injury incidence, burden, anatomical region, and type, to assess their impact on athletes’ health and availability to play. Methods: A prospective cohort study was conducted monitoring injuries via an online form filled in by each team’s medical department, categorizing player position, as well as injury type, location, and severity. Incidence and burden were calculated per 1000 player-match-hours. Results: The overall injury incidence was 54.4 injuries per 1000 player-match-hours (95% CI 30.3–96.2). Forwards had higher injury incidence than backs. Lower limbs were the most affected locations—29 injuries/1000 h of exposure (95% CI 13.7–52.0). Sprain/ligaments injuries were the most common type of injury (14.4 injuries/1000 h (95% CI 3.7–30.0), followed by muscle injuries (8.1 injuries/1000 h 95% CI 4.9–11.5). Discussion: Injury incidence was higher than what is reported in amateur competitions and lower than for professionals. Injury severity was similar to that in other studies. Our study suggests a lower injury burden than in other semi-professional and elite leagues. These findings highlight the need for targeted injury prevention strategies in the Portuguese Rugby Union to increase athletes’ availability by decreasing injury incidence and/or burden. Further research with broader participation and training injury data is needed.

## 1. Introduction

Rugbyis an emerging sport, with around 8.46 million players around the world—an increase of 11% in global participation in 2023 compared to 2022—and is played across 132 countries [1]. As it is characterized by high physical demands with frequent player-to-player impact and bouts of high-intensity activity, it has one of the highest injury incidence rates of all sports at the amateur and elite levels [2].

A meta-analysis analyzing surveillance on match injuries in elite rugby male players reported 91 match injuries per 1000 h of exposure (95% confidence interval (CI), 77–106) [3]. Another meta-analysis studied injuries in an amateur male rugby union, finding a different rate of 46.8 (95% CI, 34.4–59.2) match injuries per 1000 h of player exposure [4].

The literature is scarce on semi-professional rugby. No meta-analyses or systematic reviews on injury surveillance at this level were found, but a prospective cohort observational study analyzing four seasons in the Welsh Premiership senior men’s rugby squad during four seasons reported 99.8 injuries per 1000 player-match-hours (PMH) (95% CI, 87.3–114.0), which is a similar, but higher, number than that in professional rugby [5].

In 2023, the Portuguese National Squad made its second appearance at the World Cup, achieving the first win ever at this level and receiving Rugby Union public media and attention. “Divisão de Honra” is the Portuguese first tier, a semi-professional competition, where each team has professional players playing full-time, players with part-time jobs, and amateur players completing the squad. It is composed of 10 teams with 18 games per team, 80 min per game. After the end of the regular season, six teams qualify for the playoffs with one-game competitions. There is only a study about injury surveillance in Portugal in the “Divisão de Honra” 2017/2018 season, reporting 37.92 (95% CI: 30.71–46.33) injuries per 1000 PMH [6].

The present research aimed to gather data on the match injuries sustained by first-tier Rugby Union players during the 2022/2023 season to help promote player welfare and performance. Injury epidemiology studies are important to all those in rugby communities, including medical departments, coaches, and players. These data are essential for implementing injury prevention strategies.

## 2. Materials and Methods

### 2.1. Study Design, Participants, and Data Collection

A prospective observational cohort study (Supplementary File S1) of senior male Rugby Union players in Portugal’s top tier during the 2022/2023 season (from September 2022 to May 2023) was conducted. All 10 teams’ medical departments were invited to participate in this study, and informed consent was obtained from the players. This study was ethically approved (Regional Health Administration of Centro). Invitation to participate was sent by e-mail to every head of the medical department registered with the Portuguese Rugby Federation. Injuries were reported through an online form following “Strengthening the Reporting of Observational Studies in Epidemiology—Sports Injury and Illness Surveillance” (Table 1) [7]. The form requested information on player position, as well as injury date, type, anatomical location, and if it was a contact or non-contact injury.

### 2.2. Injury Definition

Injury was defined as “any physical complaint sustained by a player during a match which prevented the player from taking a full part in training and/or match play for more than a day following the day of injury” [8]. Time-loss severity is reported as the number of days lost to training and matches. All injuries were followed until the end of the season. If the player did not return to competition before this date, the severity was calculated according to medical department experience regarding the injury.

Injury severity ranged from slight (0–1 day’s absence), mild (4–7 days’ absence), moderate (8–28 days’ absence), to severe (over 28 days’ absence) [9,10].

Match exposure was calculated for player-match-hours [number of players (15) × number of games × game duration (80 min = 1.33 h)]. No allowances were made for exposure time for players removed (yellow or red cards), as in other studies in the literature [5,10].

### 2.3. Statistical Analysis

Injury incidence was calculated as the number of injuries per 1000 player-match-hours (number of injuries/match exposure) × 1000 with a 95% CI. Injury burden was calculated as days lost per 1000 PMH (total days lost/exposure hours) × 1000 with 95% CI. The 95% CI was calculated using bootstrapping, as suggested by Williams et al. to offer solid estimates of uncertainty for incidence and burden rate values [11]. Significant differences in values for incidence and severity were assessed by comparing non-overlapping 95% confidence intervals for the variables.

Injury data were collected using Microsoft^®^ Excel^®^ for Microsoft 365 MSO (Version 2408 Build 16. 0. 17928. 20114) 64-bit and statistical analysis was performed with IBM SPSS Statistics [version 29.0.2.0 (20)]. Shapiro–Wilk tests were used to check if there was a normal distribution of the variables or not.

## 3. Results

Only 5 of the 10 teams (50%) agreed to participate in this study for the 18 games per team during the regular season, 3 of which did not reach the playoffs. Two teams reached the playoffs, with one losing in the quarterfinals and the other in the semifinals. Total match exposure for all teams reporting injuries was 1855.35 h (forwards 989.52 h; backs 865.83 h). A total of 101 injuries were reported during the season; there were more injuries in forwards than in backs (64 vs. 38). The total injury incidence was 54.4 (95% CI, 30.3–96.2) per 1000 PMH. Injury incidence was higher in forwards at 64.4 injuries per 1000 PMH (95% CI, 38.7–103.4) than in backs at 43.9 injuries per 1000 PMH (95% CI, 15.9–86.8) (Table 1). Injury burden was 1390.4 days lost/1000 PMH(1605.9 in forwards and 1144.1 in backs). There was no statistically significant difference in injury incidence or burden according to player position (*p* = 0.798).

**Figure 1:**
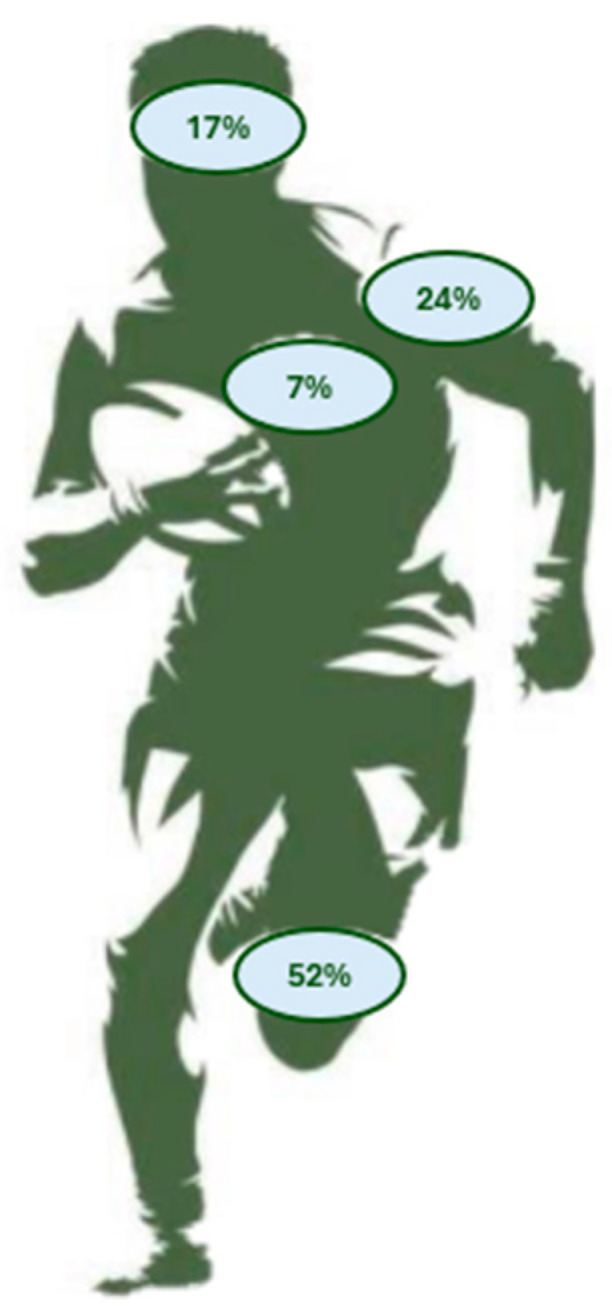
Injuries frequency according to anatomical location.

The lower limb was the most affected anatomical location, with 52% of the injuries and an incidence of 29.0 injuries per 1000 PMH (95% CI 13.7–52.0), followed by upper limb injuries, representing 24% of the total injuries (incidence of 11.6/1000 PMH—95% CI 2.6–27.2), head/neck representing 17% (10.8 injuries/1000 PMH—95% CI 7.45–14.1), and trunk representing 7% (3.9 injuries/1000 h—95% CI 2.6–27.2). Figure 1 reports injury frequency according to anatomical location.

The mean injury severity was 25.05 days (standard deviation 42.8), which was similar between positions (25.5 vs. 24.8 for forwards and backs; *p* = 0.744). For the reported injuries severity, 1 (1%) was slight, 30 (29.8%) were mild, 49 (49%) were moderate, and 21 (20.8%) were severe. Sprain/ligament injuries were the type of injury with the highest incidence, with 14.4 injuries per 1000 PMH (95% CI 3.7–30.0), followed by muscle injuries, with 8.1 injuries per 1000 PMH (95% CI 4.9–11.5). Sprain/ligament injuries had the highest burden, with 590.4 (95% CI 112.4–1068.3) days lost per 1000 PMH.

There were no significant differences observed in the injury incidence and burden between backs and forwards across anatomical location (Table 2) and type of injury (Table 3), as indicated by the overlapping 95% CI. Contact injuries were more frequent [71.3% of the injuries (*n* = 72)]. We found no statistically significant difference in the injury burden between players who sustained contact injuries and those who did not (*p* = 0.191).

All data collected are summarized in Table 2, Table 3 and Table 4 for injury incidence, severity, burden, type, and anatomical location with 95% CI.

## 4. Discussion

The primary goal of this study was to evaluate the time lost to injuries in the first tier of the Portuguese Rugby Union. Only 50% of the teams agreed to participate in this study; injury incidence was 54.4 injuries per 1000 PMH. This incidence rate was higher than what was reported in the 2017/2018 season for the same division (37.92 95% CI 30.71–46.33) [6] This number falls between the reported injury incidence for amateur competitions (46.8 per 1000 PMH 95% CI: 34.4–59.2) [3] and elite ones (91 injuries per 1000 PMH95% CI: 77–106) [4].

The variance in injury incidence could potentially be attributed to amateur players and medical departments being less likely to report minor injuries compared to those in a semi-professional league, which have more focus on injury management and have a great need for faster return to sport. Furthermore, the semi-professional structure of the “Divisão de Honra”, comprising both professional and amateur players, may also have contributed to these injury rates, bringing variations in player experience and physical readiness, possibly influencing risk exposure and injury susceptibility. However, further investigation is needed to draw definitive conclusions.

The injury severity was comparable to that in other studies, with the mean time lost per injury being 25 days, aligning with previously reported averages in Rugby Union injury research [3,4,5,6]. Our study found a lower overall injury burden (1390.4 days lost/1000 PMH) than in other semi-professional (3148.8 days/1000 PMH) and elite (2692 days/1000 PMH) rugby studies, indicating potentially fewer severe injuries or quicker recovery times within our cohort [3,5]. Sprain/ligament injuries had the highest incidence and highest burden, as found in other amateur, semi-professional, and elite studies [4,5,10]. According to the literature, most injuries (71%) were due to contact; nonetheless, the injury burden was not significantly between contact and non-contact injuries.

The implementation of prevention programs as “Activate Programme” seem to reduce injury incidence, producing a 40% reduction in injuries in the higher affected anatomical regions (lower limbs) and a 60% reduction in concussion incidence [12]. This study reinforces the need for implementing this kind of program in the athlete’s routine.

## 5. Limitations

As only half of the teams participated in this study, this potentially limits the extrapolation of the findings. A larger sample size would have strengthened the representativeness of the results. Training injuries and non-injury-related absences were not recorded, potentially affecting the overall assessment of injury burden.

The voluntary nature of participation may have introduced selection bias, as teams with higher injury rates might have been more likely to participate.

## 6. Conclusions

Our findings highlight injury epidemiology in Portuguese top-tier rugby. This study’s findings with what has been reported for the sport. It emphasizes the need for targeted injury prevention strategies focusing on improving player availability and safety; nonetheless, further research with broader participation is needed to obtain a more comprehensive picture.

Our study found a higher injury incidence and burden for lower limbs, mostly ligament and muscle injuries, demonstrating the relevance of implementing prevention programs focused on muscles on this region in order to increase player match availability.

In our study, training injuries were not considered as teams had limitations in injury reporting and accountability for training exposure (at the semi-professional level, amateur players may miss training sessions because of work, school, family, or other issues). However, training injuries have been reported in the literature to represent a small percentage of all injuries.

Future studies should aim to increase participation rates, collect data on training injuries and non-injury-related absences, and consider using more rigorous sampling methods to mitigate selection bias. These measures would enhance the generalizability and reliability of the findings.

## Figures and Tables

**Table 1:** Online form sent to every medical team (d: day; m: month; y: year).

Question	Possible Answer
Player position	Forward/back
Date of injury	dd/mm/yyyy
Anatomical region	Head/neck; upper limb; lower limb; trunk/abdomen
Type of Injury	Abrasion; concussion; fracture; hematoma/contusion/bruise; laceration; lesion of meniscus, cartilage or disc; muscle rupture/tear/strain/cramps; sprain/ligament injury; tendon injury/rupture/tendinopathy/bursitis; other
Contact injury	Yes/no
Return to play date	dd/mm/yyyy

**Table 2:** Match time-loss injury incidence, mean severity, and burden. CI: confidence interval; d: days.

	Total Injuries	Injury Incidence (95% CI)	Mean Severity (d)	Injury Burden (95% CI)
Match injuries	101	55.9 (30.3–96.2)	25.0	1390.4 (610.3–2337.1)
Forwards	63	64.4 (38.7–103.4)	24.8	1605.9 (768.2–3004.4)
Backs	38	43.9 (15.9–86.8)	25.5	1144.1 (429.9–2019.3)

**Table 3:** Match time-loss injury incidence and burden by anatomical location and by position. CI: confidence interval.

	Injury Incidence (95% CI)			Injury Burden (95%CI)		
Match Injury	All Players	Forwards	Backs	All Players	Forwards	Backs
Head/Neck	10.8 (7.5–14.1)	14.0 (6.7–19.2)	13.0 (3.3–27.4)	164.5 (105.4–222.2)	198.3 (81.2–294.7)	137.8 (67.0–178.8)
Upper Limb	11.6 (2.6–27.2)	15.4 (7.0–26.1)	9.5 (0–28.6)	168.7 (28.9–344-0)	204.6 (54.2–355.1)	127.7 (0.0–383.1)
Lower Limb	29 (13.7–52.0)	29.7 (14.3–54.8)	28.3 (11.5–47.5)	979.2 (427.6–1685.3)	1056.7 (373.6–2322.0)	890.7 (342.9–1752.3)
Trunk	3.9 (0.6–8.9)	11.6 (2.6–27.2)	0.0 (0.0–0.0)	78.0 (15.6–152.6)	146.2 (29.2–286.1)	0.0 (0.0–0.0)

**Table 4:** Match time-loss injury incidence and burden by type. CI: confidence interval.

	Injury Incidence (95% CI)			Injury Burden (95% CI)		
	All Players	Forwards	Backs	All Players	Forwards	Backs
Abrasion	0.53 *(*0.0–1.6)	1.0 *(*0.0–3.0)	0.0 *(*0.0–0.0)	2.6 *(*0.0–7.9)	4.9 *(*0.0–14.8)	0.0 *(*0.0–0.0)
Concussion	4.9 *(*2.6–8.3)	5.1 *(*0.9–10.4)	4.8 *(*1.2–8.4)	83.2 *(*30.6–141.1)	68.4 *(*8.5–156.0)	87.7 *(*16.7–175.4)
Fracture	4.4 *(*2.1–6.7)	5.1 *(*2.1–8.2)	3.6 *(*1.2–6.0)	126.1 *(*44.8–228.1)	151.9 *(*58.5;273.1)	84.6 *(*16.7–159.8)
Hematoma/contusion/bruise	7.1 *(*1.0–16.2)	9.3 *(*0.9–23.0)	4.7 *(*1.1–8.4)	83.3 *(*7.8–201.3)	129.7 *(*5.2;339.6)	26.4 *(*6.6–46.3)
Laceration	4.2 *(*1.1–7.3)	6.9 *(*2.0–11.8)	1.1 *(*0.0–3.2)	27.0 *(*6.1–49.9)	44.0 *(*11.5–76.6)	6.6 *(*0.0–19.7)
Lesion of meniscus, cartilage, or disc	2.8 *(*0.0–7.2)	3.13 *(*0.0–9.4)	2.4 *(*0.0–4.8)	133.7 *(*0–369.8)	220.3 *(*0.0–661.0)	30.3 *(*0.0–88.8)
Muscle rupture/tear/strain/cramps	8.1 *(*4.9–11.5)	11.0 *(*6.2–15.9)	6.0 *(*1.2–10.7)	187.4 *(*90.6–271.9)	285.6 *(*169.3–406.4)	65.8 *(*0.0–134.7)
Other	1.7 *(*0.0–3.9)	2.1 *(*0.0–4.2)	2.1 *(*0.0–4.2)	23.9 *(*0.0–58.5)	39.1 *(*0.0–103.8)	7.3 *(*0.0–21.9)
Sprain/ligament injury	14.4 *(*3.7–30.0)	13.5 *(*3.0–28.2)	15.4 *(*4.3–32.1)	590.4 *(*112.4–1068.3)	564.1 *(*98.2–1268.0)	542.8 *(*68.5–1282.4)
Tendon injury/rupture/tendinopathy/bursitis	5.0 *(*0.6–10.0)	7.3 *(*0.0–15.7)	6.0 *(*0.0–15.5)	85.2 *(*8.4–199.4)	99.2 *(*0.0–239.1)	60.6 *(*0.0–150.4)

## Data Availability

The raw data supporting the conclusions of this article will be made available by the authors on request.

## Supplementary Materials

The following supporting information can be downloaded at: https://www.mdpi.com/article/10.3390/ijerph21091191/s1, File S1: STROBE Statement—Checklist of items included in reports of cohort studies

## References

[1] World Rugby Global Rugby Participation Increasing Ahead of Rugby World Cup 2023. https://www.rugbyworldcup.com/2023/news/836825/global-rugby-participation-increasing-ahead-of-rugby-world-cup-2023.

[2] Brooks J.H.M., Kemp S.P.T. (2008). Recent Trends in Rugby Union Injuries. Clin. Sports Med..

[3] Williams S., Robertson C., Starling L., McKay C., West S., Brown J., Stokes K. (2022). Injuries in Elite Men’s Rugby Union: An Updated (2012–2020) Meta-Analysis of 11,620 Match and Training Injuries. Sports Med..

[4] Yeomans C., Kenny I.C., Cahalan R., Warrington G.D., Harrison A.J., Hayes K., Lyons M., Campbell M.J., Comyns T.M. (2018). The Incidence of Injury in Amateur Male Rugby Union: A Systematic Review and Meta-Analysis. Sports Med..

[5] Evans S.L., Davis O.E., Jones E.S., Hardy J., Owen J.A. (2022). Match and training injury risk in semi-professional rugby union: A four-year study. J. Sci. Med. Sport.

[6] Cruz-Ferreira A.M., Rodrigues J.C., Paulo V., Cruz-Ferreira E.M., Moreira F. (2023). The burden of match injuries in Portuguese senior male club rugby union: A cohort prospective study. Sports Exerc. Med. Switz..

[7] Bahr R., Clarsen B., Derman W., Dvorak J., Emery C.A., Finch C.F., Hägglund M., Junge A., Kemp S., Khan K.M. (2020). International Olympic Committee consensus statement: Methods for recording and reporting of epidemiological data on injury and illness in sport 2020 (including STROBE Extension for Sport Injury and Illness Surveillance (STROBE-SIIS)). Br. J. Sports Med..

[8] Fuller C.W., Ekstrand J., Junge A., Andersen T.E., Bahr R., Dvorak J., Hägglund M., McCrory P., Meeuwisse W.H. (2006). Consensus statement on injury definitions and data collection procedures in studies of football (soccer) injuries. Br. J. Sports Med..

[9] Fuller C.W., Molloy M.G., Bagate C., Bahr R., Brooks J.H.M., Donson H., Kemp S.P.T., McCrory P., McIntosh A.S., Meeuwisse W.H. (2007). Consensus statement on injury definitions and data collection procedures for studies of injuries in rugby union. Br. J. Sports Med..

[10] Brooks J.H.M., Fuller C.W., Kemp S.P.T., Reddin D.B. (2005). Epidemiology of injuries in English professional rugby union: Part 1 match injuries. Br. J. Sports Med..

[11] Williams S., Shaw J.W., Emery C., Stokes K.A. (2024). Adding confidence to our injury burden estimates: Is bootstrapping the solution?. Br. J. Sports Med..

[12] Attwood M.J., Roberts S.P., Trewartha G., England M.E., Stokes K.A. (2018). Efficacy of a movement control injury prevention programme in adult men’s community rugby union: A cluster randomised controlled trial. Br. J. Sports Med..

